# Supplementary material for: Extending Janus lectins architecture: Characterization and application to protocells
Source: Comput Struct Biotechnol J. 2022 Nov 7;20:6108–19. doi: 10.1016/j.csbj.2022.11.005 (PMC9668655; doi:10.1016/j.csbj.2022.11.005)
Supplement: Supplementary data 1 [file mmc1.pdf]

Extending Janus lectins architecture, and application for protocell labeling

Simona Notova [a], Lina Siukstaite [b,c], Francesca Rosato [b,c], Federica Vena [d], Aymeric Audfray [e], Nicolai Bovin [f], Ludovic Landemarre [d], Winfried Römer [b,c,g], Anne Imberty [a*]

MSSVQTAATSWGTVPSIRVYTANNGKITERCWDGKGWYTGAFNEPGDNVSVTSWL
VGSAIHIRVYASTGTTTTEWCWDGNGWTKGAYTATNPNGELLSSMSLRRGIYHIEN
AGVPSAIDLKDGSSSDGTPIVGWQFTPDTINWHQLWLAEPIPNVADTFTLCNLFSGTY
MDLYNGSSEAGTAVNGWQGTAFTTNPHQLWTIKKSSDGTSYKIQNYGSKTFVDLVN
GDSSDGAKIAGWTGTWDEGNPHQKWYFNRM-

Supplementary Figure 1: Amino acid sequence of Janus lectin RSL-MOA. *The lectin consists of monomer RSL at the N-terminus (blue) linked through eight AA linker (red) to β-trefoil domain of MOA at C-terminus (green).*

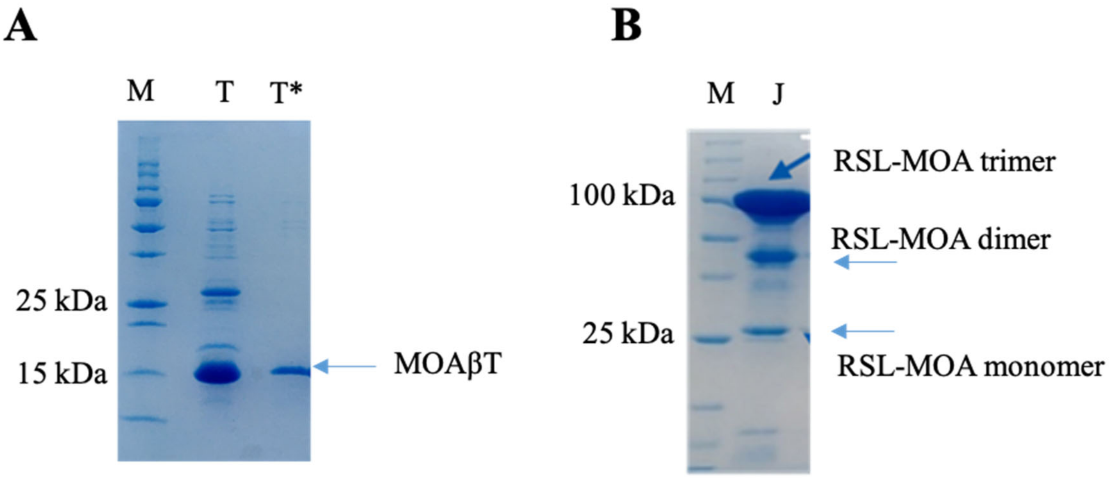

Supplementary Figure 2: SDS PAGE analysis of MOAβT and RSL-MOA. *The protein samples were analyzed under denaturing conditions on 12% polyacrylamide gel. M-protein marker, J-Janus lectin, T- MOAβT, T*- MOAβT cleaved by TEV protease. A) MOAβT with the estimated size of 17.2 kDa appears as monomeric and the impurities were eliminated during TEV cleavage and subsequent purification of protein. B) Janus lectin RSL-MOA is resistant to denaturation conditions and on the gel appears as a monomer (28 kDa), dimer (56 kDa), and trimer (83 kDa) whereas the trimer is the most abundant.*

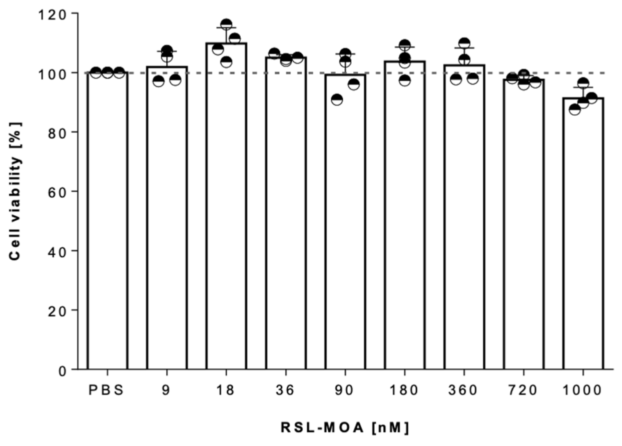

Supplementary Figure 3: Viability of H1299 cells upon treatment with RSL-MOA*. Evaluation of cell viability and proliferation following the addition of RSL-MOA in a standard cell proliferation assay (MTT) at 24 h post-treatment. The percentage of viability for treated samples is compared to control treatment with PBS. The results indicate that cell viability and proliferation are preserved when RSL-MOA is added to cells in the range 0 – 1 μM. Individual values from four independent experiments are shown, n = 4.*
